# Residues of Priority Organic Micropollutants in *Eruca vesicaria* (Rocket) Irrigated by Reclaimed Wastewater: Optimization of a QuEChERS SPME-GC/MS Protocol and Risk Assessment

**DOI:** 10.3390/foods14172963

**Published:** 2025-08-25

**Authors:** Luca Rivoira, Simona Di Bonito, Veronica Libonati, Massimo Del Bubba, Mihail Simion Beldean-Galea, Maria Concetta Bruzzoniti

**Affiliations:** 1 Department of Chemistry, Università degli Studi di Torino, Via Pietro Giuria 7, 10125 Torino, Italy; simona.dibonito@unito.it (S.D.B.); veronica.libonati@edu.unito.it (V.L.); 2 Department of Chemistry “Ugo Schiff”, Università degli Studi di Firenze, Via della Lastruccia 13, 50019 Sesto Fiorentino, Italy; massimo.delbubba@unifi.it; 3 Faculty of Environmental Science and Engineering, Babeş-Bolyai University, 30 Fântânele Street, 400294 Cluj-Napoca, Romania; simion.beldean@yahoo.com

**Keywords:** QuEChERS, SPME–GC/MS, polycyclic aromatic hydrocarbons, polychlorinated biphenyls, rocket (*Eruca vesicaria*), reclaimed wastewater, food safety

## Abstract

The increasing use of reclaimed wastewater in agriculture raises growing concerns about the accumulation of priority organic micropollutants in edible crops. In this study, we developed and validated a novel QuEChERS–SPME–GC/MS method for the simultaneous determination of 15 polycyclic aromatic hydrocarbons (PAHs), 3 nitro-PAHs, and 14 polychlorinated biphenyls congeners in *Eruca vesicaria* (rocket) leaves. The method was optimized to address the matrix complexity of leafy vegetables and included a two-step dispersive solid-phase extraction (d-SPE) cleanup and aqueous dilution prior to SPME. Validation showed excellent performance, with MDLs between 0.1 and 6.7 µg/kg, recoveries generally between 70 and 120%, and precision (RSD%) below 20%. The greenness of the protocol was assessed using the AGREE metric, yielding a score of 0.60. Application to rocket samples irrigated with treated wastewater revealed no significant accumulation of target pollutants compared to commercial samples. All PCB and N-PAH congeners were below detection limits, and PAH concentrations were low and mostly limited to lighter compounds. Human health risk assessment based on toxic equivalent concentrations confirmed that estimated cancer risk (CR) values 10^−9^–10^−8^ were well below accepted safety thresholds. These findings support the safe use of reclaimed water for leafy crop irrigation under proper treatment conditions and highlight the suitability of the method for trace-level food safety monitoring.

## 1. Introduction

Water scarcity and climate change are driving an increased reliance on reclaimed wastewater for agricultural irrigation [1]. Reusing treated wastewater in farming offers sustainability benefits: it conserves limited freshwater resources and provides a continuous, nutrient-rich water supply for crops, even in arid regions [2]. Indeed, agriculture already constitutes the largest consumer of global freshwater (about 70% of withdrawals), and in some water-scarce countries, reclaimed water now contributes significantly (e.g., ~20% in Tunisia) to irrigation, boosting food production while saving potable water [2]. However, alongside these benefits come challenges for food safety. In 2020, the EU Regulation 2020/41 was approved to promote water reuse in agriculture. The Regulation, besides setting minimum quality parameters to be monitored, requires a risk management plan to be developed to ensure safe water reuse and the safety of crops. In fact, micropollutants (both organic and inorganic) potentially present in treated wastewater or in the environment can be introduced into agro-ecosystems and accumulate in edible crops [3].

Among them, priority organic pollutants, such as polycyclic aromatic hydrocarbons (PAHs), polychlorinated biphenyls (PCBs), and nitro-PAHs (N-PAHs), are still of concern because they are persistent compounds with known human health risks [3,4]. PAHs are ubiquitous combustion byproducts found in air, water, and soil; sources include vehicle exhaust, industrial emissions, and agricultural burning, as well as irrigation with inadequately treated wastewater and application of sewage sludge [3]. Several PAHs (e.g., benzo[a]pyrene and related high-molecular-weight congeners) are classified as probable human carcinogens by the U.S. EPA and IARC [5]. PCBs, although banned decades ago, remain pervasive as legacy pollutants—they are highly lipophilic, resistant to degradation, and tend to bioaccumulate in food chains [6]. Additionally, thirteen PCB congeners exhibit dioxin-like toxicity and are among the most hazardous, prompting strict regulatory limits in foods [6]. Likewise, nitro-PAHs formed from atmospheric reactions (for instance, diesel exhaust emissions) are direct-acting mutagens, and several of them are even more carcinogenic than their parent PAHs [4]. Owing to their harmful properties, these classes of micropollutants are routinely monitored in environmental matrices. However, their behavior in agricultural reuse systems and potential long-term impacts on crop safety remain less studied and under-investigated, representing a notable knowledge gap [2].

Contamination of food crops with PAHs, PCBs, and related compounds is a significant route of human exposure and a growing public health concern [2]. In particular, leafy vegetables are recognized to accumulate these pollutants at higher levels than other crop types [3]. Indeed, due to their large surface area and often waxy or hairy leaf surfaces, leafy greens could adsorb micropollutants such as PAHs, PCBs, and N-PAHs from atmospheric deposition and irrigation water, whereas root and fruit vegetables generally show lower uptake [3,6]. For example, some surveys have found that total PAH concentrations in leafy vegetables can exceed those in root vegetables by an order of magnitude under polluted conditions [3]. Similarly, PCBs and organochlorine residues tend to be detected at slightly higher levels in leafy greens (spinach, cabbage, lettuce, etc.) than in tubers or fruits from the same farms [6]. This heightened accumulation raises still higher safety issues because many leafy vegetables are consumed raw, without any cooking step that can reduce the contaminant load. Even if residue levels are relatively low, chronic dietary intake is problematic: epidemiological assessments have linked consumption of vegetables grown on contaminated water or soil to enhanced cancer risk [6]. For instance, Khillare et al. observed that the consumption of raw leafy produce grown near a thermal power plant (a source of PAHs) was associated with increased lifetime carcinogenic risk [7]. Although measured PCB concentrations in vegetables are often below regulatory maximum limits, the presence of these persistent pollutants could pose health risks upon long-term consumption of contaminated greens [6]. These findings underscore the need for vigilant monitoring of priority pollutants in vegetables, especially as the use of reclaimed water in agriculture becomes more widespread, with the aim to ensure that wastewater-irrigated crops consumed raw like lettuce, spinach, and rocket are safe for consumers.

Within this context, *Eruca vesicaria* (commonly known as rocket or arugula) is an important but under-studied crop. Rocket is a fast-growing leafy Brassica commonly included in salads and cultivated globally, prominently in Mediterranean diets. Rocket is often grown in open fields or soilless systems that might utilize nonconventional water sources, including tertiary-treated wastewater, as part of sustainable agriculture practices. Notably, *E. vesicaria* can be a particularly challenging matrix for organic micropollutant residue analysis. In fact, like other leafy greens, rocket leaves have high water content and abundant pigments (chlorophylls, carotenoids) and polyphenolic compounds, all of which can interfere with analytical detection of trace organic micropollutants [8]. The intense green color and pigment load in rocket extracts can cause matrix effects (e.g., GC–MS signal suppression or instrument fouling), unless extensive clean-up is performed. Probably based on these premises, there is a lack of published analytical protocols for the monitoring of organic residues tailored for rocket. Most existing multi-residue methods for organic pollutants in vegetables have focused on more traditional crops (such as lettuce [9], tomato [10], carrot [11] or olives [12]), but their performance on rocket has not been systematically evaluated yet. This gap highlights the need to develop optimized methods that can reliably detect PAHs, PCBs, and N-PAHs in rocket leaves at very low concentration levels which nevertheless can represent a risk for health.

In recent years, several sample preparation approaches have been explored for organic micropollutant analysis. These include microextraction and sorptive techniques, such as solid phase microextraction (SPME), and Quick, Easy, Cheap, Effective, Rugged, and Safe (QuEChERS) approaches. SPME is solvent-free and can minimize matrix effects [13], but cannot be directly used on solid matrices. On the other hand, QuEChERS is a simple and tunable technique for solid samples that drastically reduces solvent usage and sample handling. Although QuEChERS is usually based on acetonitrile extraction [14], direct injection of an acetonitrile extract into a GC–MS non-polar column may still result in baseline noise and peak splitting [15]. Hence, an integration of SPME into QuEChERS workflows for pollutant analysis could join the benefits of both techniques, as demonstrated by the study of Al-Alam and co-workers [16] for honey analysis.

Based on the above-mentioned premises, in this study we present an innovative analytical protocol for the determination of priority organic micropollutants in *Eruca vesicaria* (rocket) leaves, using an optimized QuEChERS sample preparation combined with SPME–GC/MS. We focus on a representative list of 15 PAHs, 3 nitro-PAHs, and 14 PCB congeners (including dioxin-like ones). The method’s performance was thoroughly evaluated, directly in rocket matrix, in terms of extraction efficiency, detection limits, and accuracy, filling the current gap in targeted protocols for this leafy vegetable. Additionally, a greenness assessment of the developed protocol was performed to evaluate its compliance with the twelve principles of green analytical chemistry, using the AGREE metric as a quantitative tool.

Finally, the validated method was applied to rocket plants irrigated with treated wastewater to assess real-world residue levels, and a human health risk assessment was performed to evaluate potential exposure risks through dietary intake of rocket, making this study one of the few combining environmental monitoring with food safety implications for this specific crop.

## 2. Materials and Methods

### 2.1. Reagents and Standards

All reagents were analytical grade or higher. Acetonitrile was used as solvent for the QuEChERS extraction and it was obtained from Sigma-Aldrich (Merck, Darmstadt, Germany). Anhydrous magnesium sulfate (MgSO_4_) and sodium chloride (NaCl) for salting-out and drying processes were also from Sigma-Aldrich. Different dispersive solid-phase extraction (d-SPE) sorbents were evaluated: primary secondary amine (PSA), endcapped C18 bonded silica (both from Agilent Technologies, Santa Clara, CA, USA), and a strong anion exchange (SAX) sorbent (quaternary amine functionalized, Supelco/Merck, Darmstadt, Germany).

Sulfuric acid (95–97%) was purchased from Honeywell (Offenbach, Germany). Ultrapure water (18.2 MΩ·cm at 25 °C) was produced in-house using a Millipore Milli-Q system.

A total of 32 target analytes, including PAHs, PCBs, and N-PAHs were selected for analysis. These include the 15 EPA priority PAHs and 14 PCB congeners, along with 3 N-PAHs (nitro-derivatives of selected PAHs). The list of all target compounds is provided in Table S1 of the Supplementary Material, including their abbreviations and main chemical properties. Individual stock standard solutions of PAHs (100 mg/L in toluene) and N-PAHs (100 mg/L, in acetonitrile or toluene) were obtained from AccuStandard (New Haven, CT, USA) and Sigma-Aldrich. A PCB mix standard (500 mg/L in dichloromethane), containing the 14 target congeners, was purchased from LGC Standards (Milan, Italy). Isotope-labeled analogs were used as surrogate standards and internal standards for quantification and recovery correction. Specifically, a mixture of deuterated PAHs (each 5 mg/L) and a labeled N-PAH (1-nitropyrene-d_9_, 5 mg/L) from Wellington Laboratories (Guelph, ON, Canada) were added to each sample as surrogate spikes, and a ^13^C_12_-labeled PCB mix (containing ^13^C_12_-PCB 28, 52, 118, 153, 180 at 2 mg/L each; AccuStandard) was used for PCB surrogates. Anthracene-d_10_ and ^13^C_12_-PCB70 were employed as internal standards for the PAH/N-PAH and PCB groups, respectively. All standard solutions were stored at −10 °C in the dark when not in use.

### 2.2. Instrumentation and Chromatographic Conditions

Analysis of the extracts was performed on a gas chromatograph coupled to a mass spectrometer (GC–MS). The system consisted of an Agilent 6890N gas chromatograph interfaced with an Agilent 5973N quadrupole MS detector (Agilent Technologies, Palo Alto, CA, USA), equipped with a Flex EST II autosampler. Analyte separation was achieved on a low-polarity fused-silica capillary column (30 m × 0.25 mm i.d., 0.25 μm film thickness, 5%-phenyl-methylpolysiloxane phase). High-purity helium (≥99.999%) was used as the carrier gas at a constant flow rate of 1.0 mL/min. The injector was maintained in splitless mode for thermal desorption of the SPME fiber (see Section 2.5) and was set to 270 °C. The GC oven temperature program and MS acquisition parameters (ionization mode, m/z acquisition ions, etc.) are summarized in Table S2 of the Supplementary Material.

The MS operated in electron impact (EI) ionization at 70 eV. Data were acquired in selected-ion monitoring (SIM) mode, targeting characteristic quantifier ions for each analyte, as reported in Table S1 of the Supplementary Material. Instrument control and data analysis were performed through Agilent ChemStation (vC.01.0x) and Agilent Mass Hunter (v10.0) software, respectively.

### 2.3. Sample Collection and Preparation

Nine rocket salad samples (*Eruca vesicaria*, also known as arugula) were collected and analyzed in this study. All rocket samples originated from controlled experimental trials performed within the framework of some EU-funded projects, ensuring the reliability and traceability of the water sources. In details, eight out of the nine samples were obtained from agricultural test fields located in Central Italy and irrigated with drinking water (RW1–4, serving as a reference control) or with treated wastewater effluents further refined with phytoremediation mixed with freshwater (1:1, RW5–8). One sample was a commercially sourced rocket salad purchased from a local retailer (C1). Each sample consisted of freshly harvested edible rocket leaves collected in summer 2023. The sample size was designed to represent both control and treated conditions in a pilot-scale study, consistent with the methodological aim of this work.

After collection, samples were processed immediately or stored at 4 °C for no more than 24 h before preparation. Prior to extraction, rocket leaves underwent a freezing drying procedure (−20 °C) to prevent analyte degradation and to obtain a representative sample. Once fully dried, the rocket material was finely ground using a ceramic mortar and pestle until a consistent powder was obtained. Samples were stored in airtight containers at −10 °C until extraction. For each batch of analyses, a laboratory blank (solvent and reagents only) was prepared alongside the rocket samples to account for any background contamination.

### 2.4. QuEChERS Extraction Protocol

The extraction of organic micropollutants from rocket samples followed a QuEChERS-based approach, optimized through preliminary experiments.

For the extraction, an aliquot of 0.07 g of freeze-dried, homogenized rocket sample was weighed into a 50 mL PTFE centrifuge tube. Ultrapure water (10 mL), magnesium sulfate (1 g) and sodium chloride (0.4 g) were added, followed by 10 mL of acetonitrile. The tube was vigorously shaken in a vortex for 5 min (300 rpm) and then centrifuged at 7871× *g* for 5 min. Within the method development, the effect of extraction pH was examined to promote the partition of interfering species (e.g., chlorophyll, carotenoids, etc.) in the aqueous phase, rather than in the organic phase (undergoing analysis), and to increase the recovery of the analytes. For this reason, the pH of the aqueous phase was adjusted to pH 4, 9, or 13 using dilute hydrochloric acid or sodium hydroxide.

After the extraction phase, an optimized amount of d-SPE sorbents (see Section 3.2.2) was added to 5 mL of the extract in a 15 mL tube, to purify the extract. The tube was shaken for 5 min and centrifuged for 10 min (7871× *g*).

A second d-SPE purification was shown to be necessary to further abate the matrix content. Hence, 3 mL of the supernatant were again transferred into a 15 mL tube, containing a fixed mass of d-SPE sorbents. The tube was vortexed for 3 min and centrifuged for 10 min (7871× *g*).

Finally, a 2 mL aliquot of the supernatant was withdrawn and further processed by SPME (see next paragraph).

### 2.5. SPME–GC–MS Optimization

For the preconcentration of the extracts, a 100 µm polydimethylsiloxane (PDMS) fiber was used. Due to the high affinity of target analytes with acetonitrile, an aliquot of the cleaned extract was diluted with water to shift the partition equilibrium toward the SPME fiber. Specifically, 1 mL of purified acetonitrile extract was diluted by 9 mL ultrapure water, in a 20 mL headspace vial. The proper dilution ratio (combining best adsorption condition with the lower sample dilution) was properly chosen, and its optimization is reported in Section 3.1.1. The SPME extraction temperature was also optimized.

This diluted sample was further fortified with the internal standard solution to yield a final concentration of 5 µg/L for each internal standard. The vial was then sealed with a PTFE-faced septum cap and was incubated for 10 min at 80 °C (orbital shaking, 225 rpm). Subsequently, the PDMS fiber was put in contact with the sample (direct contact mode, 20 mm exposition length) at 80 °C for 40 min (orbital shaking, 225 rpm). After the extraction time, the SPME fiber was withdrawn from the vial and immediately introduced into the GC injection port (280 °C) for thermal desorption. The fiber was held in the injector for 5 min to ensure complete desorption of all the compounds before and the GC-MS analysis started. Finally, the fiber underwent the cleaning/conditioning step in the system conditioner (280 °C for 10 min) to be ready for the subsequent analysis.

### 2.6. Protocol Validation

The optimized protocol was validated in terms of extraction recoveries, linearity, method detection limits and quantification limits (MDLs, MQLs) and precision.

#### 2.6.1. Extraction Recoveries

The accuracy of the method was evaluated through recovery experiments, following the SANTE/11312/2021 guidelines. A commercial rocket sample was fortified prior to extraction with known concentrations of the target ^2^H, ^13^C_12_ and d_9_ surrogates (2 µg/kg final concentration, *C*_1_) and processed through the entire extraction and analysis procedure. A non-spiked rocket sample was processed in parallel to obtain an extract aliquot, in which the same concentration of surrogates was fortified (*C*_2_) prior to GC–MS analysis. The recovery yields were calculated based on three replicates, according to the following equation:

R
e
c
o
v
e
r
y 
Y
i
e
l
d
s %=
C
1
C
2×
100

#### 2.6.2. Linearity

Linearity was evaluated through matrix-matched calibration in the range included between 2 ng/L and 90 ng/L for PAHs, between 5 ng/L and 250 ng/L for PCBs and between 20 ng/L and 1 µg/L for PCBs (1.5 orders of magnitude linearity ranges for all the analyte classes).

#### 2.6.3. MDL and MQL

The method detection limits (MDL) and method quantification limits (MQL) for the thirty-two target analytes were determined using the residual standard deviation of the calibration curve and its slope, according to the equations MDL = 3.3 × (S_y_/m) and MQL = 10 × (S_y_/m), where Sy represents the residual standard deviation and m denotes the slope of the calibration.

#### 2.6.4. Precision

Precision was assessed by replicated analysis of commercial rocket samples fortified with isotopically labeled surrogates at 2 µg/kg concentration. The intra-day precision was determined through 10 determinations on a single day of analysis. The inter-day precision was determined over the same sample through 30 determinations after 3 days.

### 2.7. Risk Analysis

To evaluate the potential health effects associated with exposure to residual micropollutants derived from rocket consumption, the concentrations of micropollutants found in rocket were used to perform a risk analysis, following the Environmental Protection Agency (EPA) international guidelines [17]. Although EPA methodologies allow risk calculations also for children, the consumption of rocket for this category is generally limited due to its characteristic bitter taste. Hence, risk assessment was here focused only on adult consumers, who represent the main population group regularly consuming rocket.

Risk characterization included the calculation of the Lifetime Average Daily Dose, *LADD*, according to the following equation

$$LADD=\frac{C\cdot IR\cdot EF\cdot ED}{BW\cdot AT}$$

where *C* is the total micropollutant concentration in the fresh rocket, *IR* is the intake rate (g/d), *EF* is the exposure frequency (d/y), *ED* the duration of exposure (y), *BW* the body weight (kg), *AT* the average time of exposure (d).

To assess cancer risk (*CR*), the probability of developing cancer due to chronic exposure, the cancer slope factor (*CSF*, mg/kg-d) was calculated through the following relation:

CR=LADD·CSF

According to EPA, a *CR* value exceeding 10^−6^ (one in a million cases) is typically considered to pose a potential health concern.

## 3. Results and Discussions

### 3.1. Optimization of SPME Conditions

#### 3.1.1. Fiber Selection and Extract Dilution

**Figure 1:**
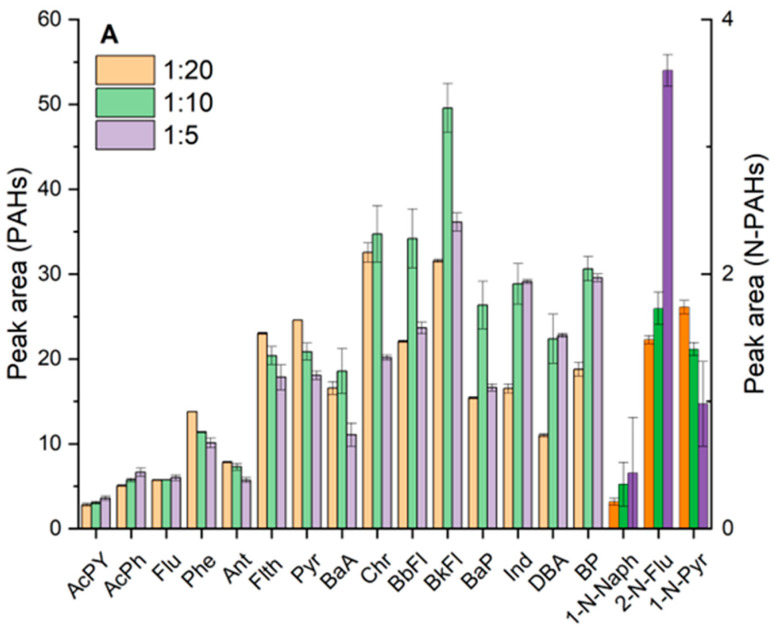

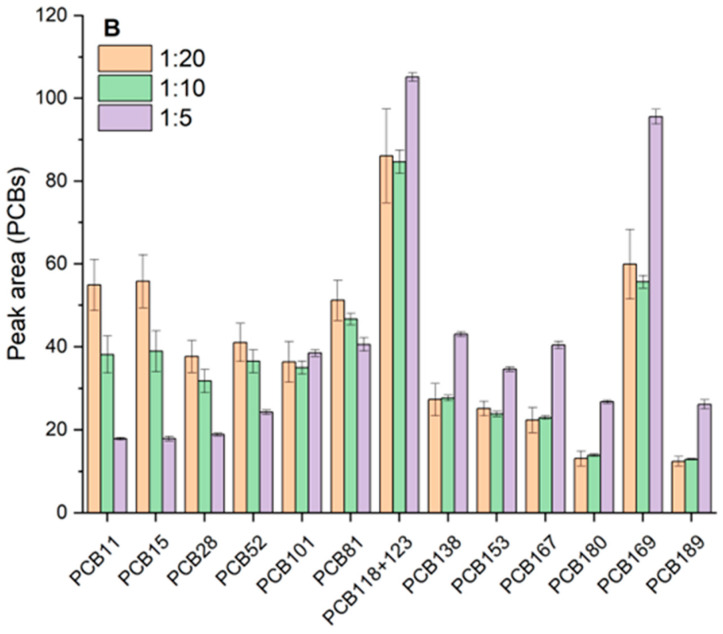
Effect of extract dilution (CH_3_CN:H_2_O) on SPME–GC/MS signal intensity for selected analytes in rocket samples: (**A**) PAHs and N-PAHs (brighter color) and (**B**) PCBs. Acetonitrile: water dilution ratios evaluated were 1:5, 1:10, and 1:20. Peak areas were normalized against internal standards.

A 100 µm PDMS SPME fiber was selected for its high affinity toward nonpolar analytes (PAHs, N-PAHs and PCBs) and robustness at GC desorption temperatures [18]. Initial trials revealed that direct SPME of CH_3_CN QuEChERS extract was ineffective due to CH_3_CN competition with analyte partitioning onto the fiber. Hence, the extract dilution with water (CH_3_CN:water ratios of 1:2, 1:5, 1:10, 1:20) was investigated, to reduce CH_3_CN content. It is important to consider that increasing the dilution of the QuEChERS extract generally improves analyte–fiber interactions by reducing the competition from organic solvent. However, excessive dilution can also compromise sensitivity by lowering the overall preconcentration factor achievable via SPME. Thus, an optimal balance must be found between extraction efficiency and final analyte detectability. In this context, the 1:2 CH_3_CN:water dilution was found to be too low: indeed, analyte partitioning onto the fiber remained stronlgy hindered, as evidenced by broadened and tailing peaks, and this condition was therefore discarded. As reported in Figure 1 (A and B), the optimal dilution among 1:5, 1:10, 1:20 depended on the analyte properties.

To what concern PAHs (Figure 1A), for very volatile 2-ring PAHs (e.g., naphthalene), extraction was nearly unaffected by dilution, with a slight improvement at higher CH_3_CN fraction (1:5). In contrast, moderately volatile 3–4 ring PAHs (e.g., phenanthrene, fluoranthene) showed poor recovery with high content of CH_3_CN. Indeed, for these last compounds, the signal was more intense at the highest water content (1:20) as water’s poor solvating power “pushed” these hydrophobic analytes onto the fiber. Interestingly, for the heaviest PAHs (5–6 rings like benzo[a]pyrene, indeno[1,2,3-cd]pyrene, etc.), an intermediate dilution (1:10) gave the highest response. Excess of water (1:20) could reduce the partition in the solvent mixture of these extremely hydrophobic compounds to efficiently diffuse to the fiber.

A similar trend was observed for PCBs (Figure 1B): lower-chlorinated congeners preferred water-rich conditions, while highly chlorinated PCBs required some CH_3_CN to remain sufficiently partitioned. Nitro-PAHs (Figure 1A) exhibited mixed behaviors—e.g., 1-nitropyrene (a heavy N-PAH)—were better extracted in the presence of higher amount of more water, whereas 2-nitrofluorene (lighter, more polar) resulted better extracted in the presence of higherth more CH_3_CN content. Balancing these results, a 1:10 dilution (10% CH_3_CN in water) was chosen as the optimum compromise that provided strong extraction efficiency across all target analytes. This dilution increased overall SPME sensitivity relative to using undiluted CH_3_CN, confirming the importance of minimizing organic solvent content for SPME of hydrophobic compounds.

#### 3.1.2. Extraction Temperature

The SPME incubation/extraction temperature was found to significantly influence extraction kinetics and equilibria. Higher temperatures (up to 80 °C) generally increased analyte diffusion and volatility, enhancing transfer to the fiber [19]. However, higher temperatures can have negative effects on target pollutants, like thermal degradation. Based on the considerations, the effect of two different incubation/extraction temperatures for SPME, 50 °C and 80 °C, were investigated (the other SPME extraction conditions are reported in Section 2.5). The above-mentioned temperatures ensured appropriate volatilization, avoiding thermal degradation [20].

**Figure 2:**
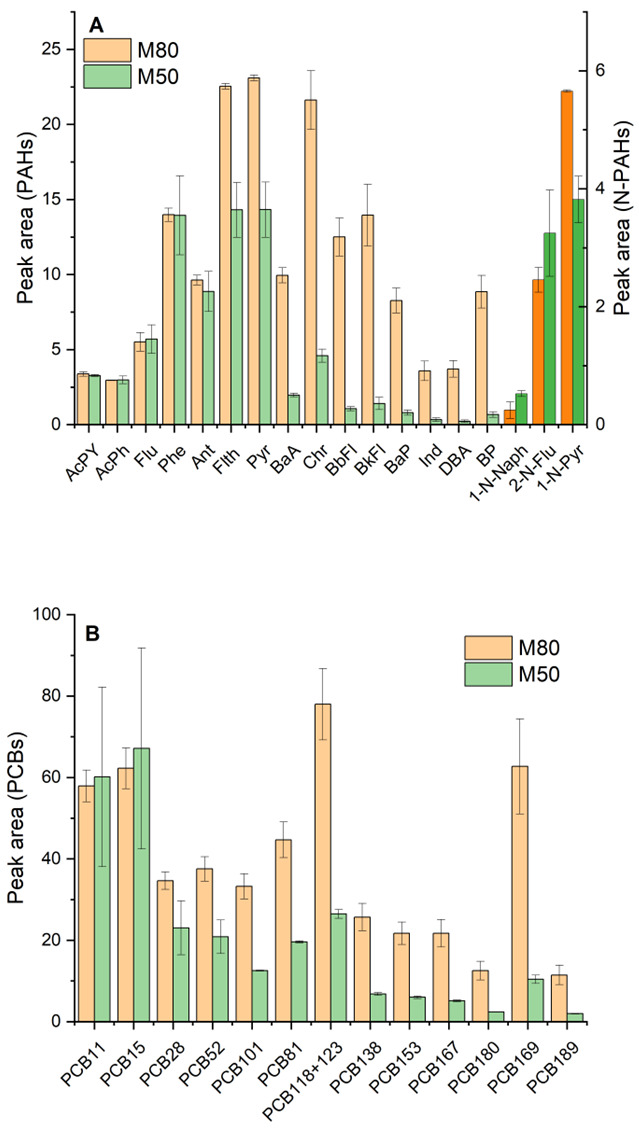
Effect of extraction temperature (50 °C vs. 80 °C) on SPME–GC/MS response for representative compounds in rocket extracts: (**A**) PAHs and N-PAHs (brighter color) and (**B**) PCBs.

As expected, raising the temperature to 80 °C markedly improved responses for most high-molecular-weight, low-volatility analytes—notably the heavier PAHs (4–6 rings), 1-nitropyrene, and the PCB congeners with ≥5 chlorine atoms (Figure 2A and Figure 2B, respectively).

At 50 °C, these compounds tended to remain in solution, limiting their uptake, whereas at 80 °C their reduced aqueous solubility and a faster diffusion drove them into the PDMS fiber in a higher extent. Conversely, for the most volatile PAHs (i.e., acenaphthylene, acenaphthene, fluorene, phenanthrene) and the lowest chlorinated PCBs, extraction yields were similar at 50 °C and 80 °C, thus meaning that these lighter compounds equilibrate quickly even at lower temperature. A few exceptions were observed: the two most polar N-PAHs (1-nitronaphthalene and 2-nitrofluorene) actually showed a slight decrease in signal at 80 °C. This behavior can be attributed to the increased solubility of nitro-PAHs in hot aqueous media, driven by the presence of the polar nitro group [21,22]. At elevated temperatures, their enhanced affinity for the aqueous phase reduces their tendency to partition onto the SPME fiber, thereby limiting the overall extraction efficiency despite the higher volatility. Additionally, prolonged high temperature might cause nitroaromatic instability [20]. Considering the overall improvement for the majority of target compounds, 80 °C was selected as the optimal incubation/extraction temperature.

In summary, 1:10 extract dilution and 80 °C incubate/extraction temperature were chosen as optimal since they jointly maximized the extraction efficiency for a broad range of analytes.

### 3.2. Optimization of QuEChERS Extraction and Cleanup

#### 3.2.1. Extraction pH

From an analytical point of view, rocket leaves are a challenging matrix with high amount of pigments (chlorophylls, carotenoids) and polyphenols that can be co-extracted with analytes of interest [23]. To minimize pigment co-extraction (hence to promote their dissolution in the acqueous phase, that is not futher analyzed and discarded) the effect of pH on the initial extraction step was explored. Acidic (pH 4) and basic (pH 9 or 13.5) conditions were tested by adjusting the aqueous phase before CH_3_CN addition. Extreme basic conditions proved most effective at partitioning green pigments into the water phase: at pH 13.5 the CH_3_CN extract’s color was dramatically less intense, indicating that much of the chlorophyll/xanthophyll content remained in the aqueous phase (Figure S1 of the Supplementary Material). This is in accordance with base-induced deprotonation and increased water-solubility of plant pigments, as well as saponification of chlorophyll’s phytol chain, which keeps it out of the organic layer [24]. Although strong base could potentially hydrolyze sensitive analytes, the PAHs and PCBs are quite stable, and the nitro-PAHs showed no significant degradation during the short extraction period. Hence, a basification of the aqueous phase at pH 13.5 gave a cleaner organic phase extract and was selected for the optimized protocol.

#### 3.2.2. Dispersive-SPE Sorbents and Cleanup Strategy

Multiple d-SPE sorbents were evaluated in one or two-step cleanups to remove residual matrix from the CH_3_CN extract. Initial single-step tests of individual sorbents (PSA, C18, SAX) showed that each could only partially reduce the residual green color. C18 was very effective at adsorbing hydrophobic pigments (e.g., chlorophyll), turning the sorbent visibly green. PSA and SAX (both anion exchangers) primarily target polar acids and did not remove much pigment on their own, since only a slight color change was observed with SAX or PSA.

Since each individual sorbent exhibited specific but limited matrix removal capabilities and none of them alone provided satisfactory cleanup, to improve overall extract purification while preserving analyte recoveries, additional adsorbent materials were explored as well as combinations of the previously tested sorbents.

In more detail, the use of graphitized carbon black (GCB), an effective adsorbent for pigments, was problematic; in fact, while GCB removed chlorophyll, it also irreversibly adsorbed almost all PAHs and N-PAHs due to the π-π interactions between the planar aromatic molecules and the GCB structure. Interestingly, PCB recoveries remained fairly high indicated that this class of compounds remained less affected by the GCB structure indicating low interaction. Despite toluene was added to the extract to occupy GCB sites [25], this operation did not improve recoveries, which remained negligible for all classes of analytes.

Alternative carbonaceous materials were also tested, namely a styrene-divinylbenzene polymer and a biochar, but they completely adsorbed all target analytes during cleanup. Natural zeolite minerals (chabazite and zeolite 13X, activated by NaCl and NaOH) were also tested to remove pigments. Even if zeolites showed some pigment removal, a poor reproducibility was observed, thus making this approach unreliable.

An oxidative cleanup using sodium hypochlorite was also additionally evaluated. While NaClO oxidates chlorophyll, it also partially oxidizes several PAHs. This confirmed that a strong oxidizer, despite its decolorizing efficacy, is incompatible with preserving target trace organic pollutants.

Based on these preliminary results, it became evident that the most promising strategy relied on the initial set of sorbents (namely, SAX, PSA, and C18) which, although only partially effective individually, offered complementary selectivity. Consequently, efforts were focused on combining these materials and applying them in sequential cleanup steps to enhance matrix removal without reducing analyte recoveries. Hence, after extraction, the CH_3_CN layer was first treated with a mixture of C18, SAX and PSA (0.09 g, 0.27 g and 0.7 g, respectively). This step significantly decolorized the extract. Hence, a second cleanup was further applied using the same d-SPE phase combination and amounts. This two-stage dispersive cleanup was effective to achieve acceptable color residual in such a complex matrix, taking into account the 1:10 dilution with water prior to analysis.

**Figure 3:**
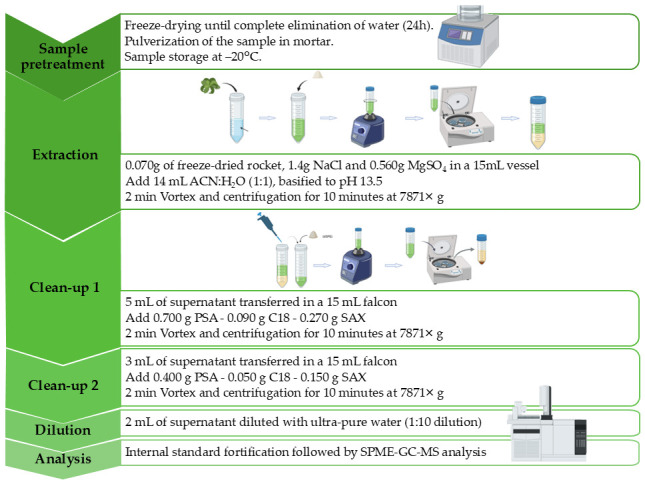
Schematic representation of the optimized analytical workflow for the determination of PAHs, PCBs, and N-PAHs in *Eruca vesicaria* leaves. The protocol integrates QuEChERS extraction with a two-step dispersive solid-phase extraction (d-SPE) cleanup, followed by SPME–GC/MS analysis after aqueous dilution of the purified extract.

The final optimized protocol is summarized in Figure 3.

Considering the overall procedure, it should be emphasized that the optimized workflow integrates two complementary principles: miniaturization through the solvent-free SPME step, which drastically reduces solvent consumption compared to traditional approaches, and the efficiency of the QuEChERS extraction in removing matrix interferences. SPME was preferred over other miniaturized approaches because it can be directly implemented into the GC–MS system, thereby reducing analysis time and operator handling steps while improving accuracy and reproducibility.

This combination ensures both analytical robustness and improved environmental sustainability (as further discussed in Section 3.4).

### 3.3. Protocol Validation

The optimized QuEChERS–SPME–GC/MS method was validated in terms of extraction recoveries, linearity, sensitivity, and precision.

#### 3.3.1. Extraction Recoveries

**Figure 4:**
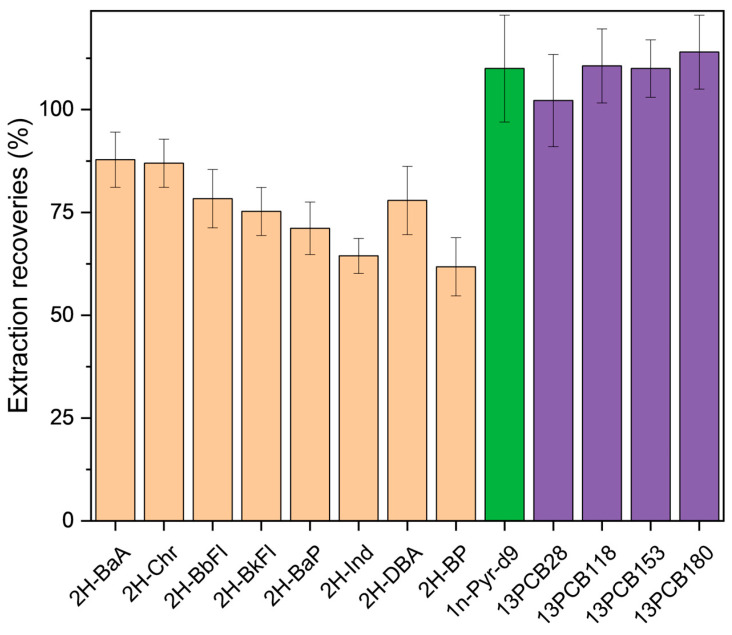
Extraction recoveries (%) of isotopically labeled surrogate compounds representing PAHs, PCBs, and N-PAHs in fortified rocket samples (*n* = 3 replicates).

Extraction efficiencies were evaluated through recovery experiments on spiked samples, using isotope-labeled surrogates for each class (see Section 2.6.1). Results, summarized in Figure 4, showed that overall recoveries for the optimized QuEChERS–SPME protocol were within 70–120% for most analytes.

Specifically, the mean surrogate recoveries for PAHs ranged from about 62% (for the heaviest PAHs) up to 88% for lighter PAHs. This trend is in accordance with previous works devoted to the extraction of PAHs from complex matrices, in which a partial decrease in extraction recoveries was observed with the increase in PAH molecular weight [10,12]. In contrast, PCB surrogate recoveries ranged more homogenously around 100–115%. 1-nitropyrene-d9 also showed a quantitative recovery.

Overall, the recovery data confirmed that the optimized protocol is effective in the extraction of the target micropollutants from rocket leaves with negligible bias.

#### 3.3.2. Linearity and Sensitivity

Calibrations for each analyte, used to evaluate linearity as well as to calculate MDLs and MQLs, were built using the matrix-matched calibration to avoid over- or underestimation potentially present due to residual matrix effects. Main figures of merits (linear equation, R^2^, MDLs and MQLs) are summarized in Table 1 for PAHs, N-PAHs and PCBs.

As reported, excellent linearity was obtained for all 32 analytes. PAHs and N-PAHs were linear from 0.1 to 90 ng/L while PCBs from 5 to 250 ng/L, with typical R^2^ values between 0.988 and 0.999.

The MDLs for individual analytes, obtained correcting detection and quantitation limits with the extraction recoveries of surrogates ranged from 0.1 to 6.7 µg/kg in the rocket (dry-weight) depending on compound. Corresponding MQLs were 0.4–20.3 µg/kg. The highest MDLs were observed for 2-N-Flu and 1-N-Pyr, which were the least sensitive target analytes.

As no specific maximum levels for PAHs, N-PAHs, or PCBs are established for fresh leafy vegetables such as rocket, the threshold values reported in EU Regulation 2023/915 for comparable matrices (e.g., dried herbs for PAHs, fat-rich foods for PCBs) were considered only as precautionary reference values. These thresholds were not applied as binding legal limits for rocket, but rather as indicative thresholds to contextualize the sensitivity and reliability of the method [26]. In detail, the maximum PAH level allowed for dried herbs is 10 µg/kg for benzo[a]pyrene and 50 µg/kg for the sum of benzo[a]pyrene, benz[a]anthracene, benzo[b]fluoranthene, and chrysene. Regarding PCBs, the regulation defines a limit of 40 µg/kg fat for the sum of six indicator congeners (not dioxin-like ones) in fat-rich foods such as vegetable oils, dairy, and meat, but no limit is established for vegetables. MDLs and MQLs here presented are well below all the previously cited thresholds, ensuring that contamination in rocket can be reliably assessed within the boundaries of current EU food safety frameworks.

#### 3.3.3. Precision

The method’s precision was assessed at two levels: intra-day and inter-day repeatability. A commercial rocket sample spiked with isotopic labeled surrogates was analyzed over 24 h (*n* = 10 samples) and on 3 separate days (*n* = 30 determinations). The relative standard deviations (RSD%) of measured concentrations are reported in Table 2.

In detail, intra-day RSDs were almost in the 5–19% range for PAHs, N-PAHs and PCBs, and inter-day RSD% were lower than 17%. Selected compounds with lower sensitivity showed RSD% up to 20% (e.g., Ind-d_12_, DBA-d_14_ or BP-d_12_). The largest variability observed was for one of the heavy PCBs (^13^C_12_-PCB-153, 23% RSD intra-day), but its inter-day RSD was only ~7% showing that the overall method reproducibility improved when averaging across days. In all cases, the RSDs remained ≤20%, fulfilling criteria for analytical quality control and method validation procedures for residues analysis in food and feed [27].

The validation results confirm that the developed procedure enables reliable quantitation of PAHs, N-PAHs, and PCBs in rocket at concentrations lower than µg/kg. To the best of our knowledge, no prior study has specifically reported the determination of PAHs or PCBs in rocket (*Eruca vesicaria*), making the present QuEChERS–SPME–GC/MS analysis the first of its kind for this matrix. Moreover, the method showed analytical performances (such as MQLs, MDLs, and recoveries) that are comparable to, or improved those reported, those reported for similar matrices including leafy vegetables (e.g., lettuce, spinach) and other plant-based foods, despite being implemented on a single quadrupole GC–MS system rather than a more selective and costly triple quadrupole instrument [28,29].

### 3.4. Greenness Evaluation and Economical Aspects

The greenness of the analytical protocol was evaluated using AGREE (Analytical GREEnness metric approach), an open-source tool developed by Pena-Pereira and collaborators [30]. AGREE quantitatively assesses compliance toward the 12 principles of green analytical chemistry, providing a unified score on a 0–1 scale. The output includes a graphical representation that highlights both the overall greenness score and the individual contributions of each principle, thereby facilitating rapid and transparent comparison among analytical methods.

**Figure 5:**
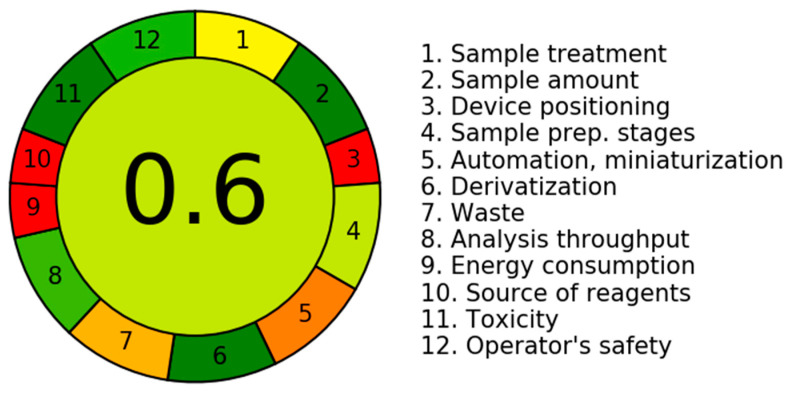
AGREE evaluation of the QuEChERS–SPME–GC/MS method developed for the determination of PAHs, PCBs, and N-PAHs in *Eruca vesicaria*. Radial segments represent the individual contributions of each green chemistry principle.

As shown in Figure 5, the optimized method here reported obtained an overall AGREE score of 0.6, which reflects a satisfactory level of environmental compatibility for a multiresidue procedure applied to a complex matrix such as rocket. High scores (green zones) were obtained for Principle 2 (minimal sample size), Principle 6 (avoidance of derivatization), Principle 8 (multi-analyte analysis), Principle 11 (use of low-toxicity reagents), and Principle 12 (operator safety), thanks to the use of miniaturized extraction (SPME), the absence of chemical derivatization, and the simultaneous detection of 32 target analytes with low solvent consumption and reduced operator exposure (QuEChERS + SPME). Moderate scores (yellow to orange segments) were observed for Principle 1 (direct techniques), Principle 4 (integration of analytical operations), Principle 5 (automation and miniaturization), and Principle 7 (waste reduction). These reflect the presence of essential offline sample handling steps (e.g., freeze-drying, centrifugation), manual extraction phases, and the unavoidable use of certain consumables and sorbents for the QuEChERS extraction. It should be mentioned that the lower scores (red zones) obtained for Principle 3 (in situ applicability), Principle 9 (use of renewable materials), and Principle 10 (energy consumption) are intrinsic limitations to most laboratory-based methods that require equipment with moderate-to-high energy demand (e.g., GC-MS) and that relies on non-renewable reagents (e.g., acetonitrile, sodium hydroxide). For these reasons, the weighting factors assigned to these three criteria were reduced to 1, compared to a weight of 2 applied to the remaining principles, which were considered more relevant to the context of this study.

Based on the above-mentioned considerations, the overall AGREE profile demonstrates that the proposed method balances analytical performance and environmental responsibility. In particular, the integration of SPME into a QuEChERS workflow contributes significantly to solvent minimization, waste reduction, and enhanced safety, positioning this method favorably among existing protocols for trace contaminant analysis in food [10].

To what concern economical aspects, compared to a traditional liquid–liquid extraction or SPE-based method, our QuEChERS–SPME protocol likely costs less per sample, owing to lower solvent and consumable use. Indeed, the main consumable, the SPME fiber, can be reused for about one hundred extractions, spreading out its cost. Additionally, reducing hazardous solvent waste is not only an environmental benefit but also cuts down on disposal costs, aligning economic and green objectives. An additional aspect of sustainability lies in the fact that the method achieves the required analytical performance using a single-quadrupole GC–MS, instead of a more resource-demanding triple-quadrupole system. This choice not only reduces instrument and maintenance costs, but also lowers overall energy consumption and CO_2_ emissions, thereby contributing to both environmental and economic sustainability.

### 3.5. Real Samples

#### 3.5.1. Analysis

The validated method was applied to the analysis of rocket leaf samples. Eight rocket samples grown up by irrigated with drinking water (chosen as a control) or treated wastewater (blended with freshwater) were analyzed to investigate the impact of reclaimed wastewater irrigation on contaminant levels. One sample of commercial rocket was also analyzed. To avoid over- or underestimation bias due to potential residual matrix or external factors, analytes were quantified by matrix-matched calibration. Procedural blanks were also run and subtracted when necessary.

As reported in Table 3, only part of the target analytes was quantified in all the samples, with a predominance of lighter PAHs and heavier compounds below the detection limits.

In details, starting from the commercial sample, eight PAHs with 2–4 rings (acenaphthylene, acenaphthene, fluorene, phenanthrene, anthracene, fluoranthene, pyrene and benzo[a]anthracene) were detected at a 5–18 µg/kg (dry weight) concentrations range. The remaining PAHs (i.e., chrysene, benzo[b/k]fluoranthenes, benzo[a]pyrene, indeno[cd]pyrene, dibenzanthracene, benzo[ghi]perylene) were not detected in the commercial rocket. Similarly, none of the PCB congeners, as well as of the N-PAHs, was detected in the commercial sample (all below method detection limits). This contamination profile, with only the more volatile PAHs present, and no heavier PAHs or PCBs, is in accordance with prior observations that leafy vegetables can accumulate airborne light PAHs (deriving from atmospheric deposition such as vehicle exhaust, urban air) [3].

Moving to the rocket plants irrigated by reclaimed water, the same group of PAHs was detected, generally at comparable levels with commercial rocket, thus meaning that these trials did not exhibit significantly higher concentrations than the commercial sample. As regards PCBs and N-PAHs, they were below MDLs also in all reclaimed-water samples. The results, therefore, suggest that irrigation with properly treated wastewater (i.e., tertiary-treated) did not lead to any substantial accumulation of the monitored organic micropollutants in rocket leaves. Our results agree with previous studies that reported minimal differences in organic contaminant residues between crops irrigated with reclaimed water versus clean water. For example, a study on greenhouse lettuce using reclaimed vs. groundwater found only low µg/kg levels of PAHs in both crops, attributable more to the atmospheric sources than to water source [11]. Similarly, a previous study from our research group evidenced that an optimized QuEChERS method recovered only trace PAHs/PCBs in tomatoes irrigated with treated water, with levels well below regulatory concern [10].

#### 3.5.2. Risk Assessment

The concentrations of PAH micropollutants observed in rocket samples were converted from dry to fresh weight using the humidity content of the fresh fruit, which was measured before the sample pretreatment protocol and was approximately 90%.

The concentrations were subsequently expressed as toxic equivalent concentrations (TEQ), using the benzo(a)pyrene (BaP)-toxic equivalent factors [31]. The CSF used was the one referred to benzo(a)pyrene, i.e., 1 mg/kg-d.

As regards the intake rate, considering that a portion of rocket is about 80 g and that the recommended daily dose of total vegetables is 180 g/d [32], an IR = 100 g/d was used. This dose corresponds to a rocket consumption of approximately 55% compared to the total vegetable consumption, and it was considered precautionary.

An EF of 365 d was considered, with a duration ED of 30 y (adult exposure) for a 70 kg BW individual.

According to the PAHs found in crops, the calculated carcinogenic risk is reported in Table 4. Since CR values are lower than 10^−6^, the consumption of these products does not represent a risk for human health. It is also possible to highlight that the risk associated with the consumption of rocket irrigated with treated wastewater (RW5–8) is not statistically different (ANOVA test, α = 0.05) from the risk associated with the consumption of rocket irrigated with drinking water (RW1–4) and the commercial one (C1).

These results were compared with those reported in the literature for PAH exposure via leafy vegetables. For instance, Khillare et al. (2012) estimated cancer risk values between 1.3 × 10^−6^ and 5.1 × 10^−5^ for populations consuming leafy vegetables (spinach, coriander, fenugreek) cultivated near a thermal power plant in Delhi, India—values that exceed acceptable limits by up to two orders of magnitude [7]. Similarly, Olatunji (2019) found CR values exceeding 1 × 10^−6^ in lettuce and cabbage grown in peri-urban areas of Nigeria with moderate environmental contamination [6]. In contrast, the risk estimates from the present study (obtained under reclaimed wastewater irrigation) are one to two orders of magnitude lower and indistinguishable from those observed in commercially available rocket. These findings strength the idea that the use of properly treated wastewater does not pose an elevated cancer risk through dietary intake of leafy vegetables, provided that pollutant levels are adequately monitored and controlled. Similar conclusions were obtained from previous studies from our research group on strawberries and olives, where no significant accumulation of PAHs, PCBs was detected in the edible fractions of crops irrigated with reclaimed water [12,33]. These consistent results across different matrices support the safe application of treated wastewater in sustainable agricultural practices, when strict water quality standards are ensured.

## 4. Conclusions

This study presents the first comprehensive investigation of priority organic micropollutants in *Eruca vesicaria* (rocket) irrigated with treated wastewater, supported by the development and validation of an innovative analytical protocol based on QuEChERS extraction combined with SPME–GC/MS. The proposed method was specifically tailored to address the complexity of the rocket matrix—characterized by abundant pigments and co-extractives—and demonstrated excellent analytical performance in terms of sensitivity (MDLs down to 0.1 µg/kg), recoveries (generally 70–120%, with the exception of benzo[ghi]perylene (65%) and indeno[1,2,3-cd]pyrene (68%)), and precision (RSD < 20% for all surrogates).

The integration of SPME into the QuEChERS workflow significantly improved method greenness, minimized solvent usage, and enhanced analyte selectivity, while the method’s overall AGREE score (0.60) confirmed its compliance to the principles of green analytical chemistry. Comparison with existing literature data on leafy and non-leafy vegetables shows that this approach is at least equivalent or superior to other reported methods, including those solely based on QuEChERS or conventional liquid–liquid extraction, even when performed on more selective instruments.

Application to field-grown rocket irrigated with treated wastewaters demonstrated no significant presence of PAHs, PCBs, or N-PAHs compared to commercial samples, with carcinogenic risk values remaining in the 10^−9^–10^−8^ range—well below health concern thresholds and markedly lower than values reported for leafy vegetables grown in contaminated environments. These results suggest that, under controlled conditions, reclaimed water can be safely used for leafy vegetable cultivation without introducing additional consumer risk related to persistent organic micropollutants, even if it is highlighted the importance of future studies to specifically address sensitive subpopulations.

Overall, this work fills a methodological gap for a relevant but under-studied crop, providing a robust and eco-compatible tool for food safety monitoring in the context of water reuse and sustainable agriculture.

## Figures and Tables

**Table 1:** Linearity (R^2^), method detection limits (MDL), and method quantification limits (MQL) for PAHs, N-PAHs, and PCBs analyzed in rocket samples using the optimized QuEChERS–SPME–GC/MS method. Values are reported on a dry weight basis (µg/kg), corrected for surrogate recovery. Calibration was performed via matrix-matched calibration to account for matrix effects.

Analites	R^2^	MDL (μg/kg)	MQL (μg/kg)
AcPY	0.999	0.3	1.0
AcPh	0.997	0.2	0.7
Flu	0.998	0.5	1.3
Phe	0.998	0.2	0.6
Ant	0.999	0.1	0.4
Flth	0.997	0.3	1.3
Pyr	0.999	0.2	0.7
BaA	0.998	0.3	0.9
Chr	0.999	0.3	1.0
BbFl	0.989	0.2	0.7
BkFl	0.997	0.4	1.3
BaP	0.996	0.4	1.1
Ind	0.985	0.3	0.7
DBA	0.994	0.3	1.9
BP	0.988	0.3	0.8
1-N-Naph	0.997	2.4	7.2
2-N-Flu	0.996	6.7	20.3
1-N-Pyr	0.995	3.4	10.2
PCB11	0.994	0.8	2.5
PCB15	0.999	0.8	2.5
PCB28	0.998	0.8	2.4
PCB52	0.997	0.9	2.6
PCB101	0.995	0.7	2.2
PCB81	0.998	0.7	2.3
PCB118 + 123	0.996	0.5	1.6
PCB138	0.997	0.4	1.2
PCB153	0.996	0.4	1.3
PCB167	0.999	0.2	0.7
PCB180	0.999	0.4	1.2
PCB169	0.997	0.5	1.7
PCB189	0.997	0.5	1.5

**Table 2:** Intra-day and inter-day precision expressed as relative standard deviation (RSD%) for selected isotopically labeled surrogate compounds in rocket samples analyzed by the optimized QuEChERS–SPME–GC/MS method. Precision was assessed over 10 replicates in a single day and 30 determinations across three non-consecutive days.

	Intraday Precision [RSD%]	Interday Precision [RSD%]
BaA-d_12_	5	10
Chr-d_12_	12	12
BbFl-d_12_	8	10
BkFl-d_12_	9	12
BaP-d_12_	12	13
Ind-d_12_	18	16
DBA-d_14_	18	15
BP-d_12_	18	17
^13 ^C_12_-PCB28	8	9
^13 ^C_12_-PCB52	9	9
^13 ^C_12_-PCB118	8	9
^13 ^C_12_-PCB153	20	7

**Table 3:** Concentrations (µg/kg dry weight ± SD) of selected PAHs detected in rocket samples irrigated with treated wastewater (RW1–8) and in a commercial rocket sample (C1). Only analytes quantified above the method quantification limit (MQL) are reported.

	RW1			RW2			RW3			RW4			RW5		
AcPy	9.0	±	1.1	7.9	±	0.5	8.0	±	0.2	5.9	±	0.2	5.9	±	2.0
AcPh	6.3	±	2.1	8.4	±	0.9	6.8	±	0.3	4.9	±	0.1	3.0	±	0.9
Flu	18.1	±	0.4	16.2	±	0.8	18.2	±	2.6	12.9	±	0.3	10.4	±	2.6
Phe	60.8	±	4.6	45.5	±	1.6	50.8	±	5.5	37.2	±	0.5	30.9	±	11.6
Ant	5.1	±	0.1	4.9	±	0.6	5.8	±	0.4	4.4	±	0.2	3.2	±	0.1
Flth	6.1	±	0.1	3.8	±	0.3	5.0	±	0.7	3.0	±	0.5	3.4	±	1.0
Pyr	5.5	±	0.1	3.3	±	0.2	4.9	±	0.6	2.8	±	0.3	3.1	±	0.8
BaA	<MQL			<MQL			<MQL			<MQL			<MQL		
	**RW6**			**RW7**			**RW8**			**C1**					
AcPy	6.1	±	0.6	11.6	±	0.2	10.2	±	0.8	11.7	±	0.1			
AcPh	3.8	±	0.0	7.9	±	0.3	7.5	±	1.6	13.3	±	0.1			
Flu	11.2	±	0.5	27.7	±	0.8	26.4	±	3.1	13.4	±	0.2			
Phe	26.0	±	2.4	77.8	±	4.9	69.0	±	5.0	14.3	±	1.3			
Ant	3.3	±	0.4	7.6	±	0.2	8.3	±	0.1	5.5	±	0.1			
Flth	1.6	±	0.4	7.6	±	0.6	6.3	±	0.1	10.4	±	0.1			
Pyr	2.1	±	0.2	6.8	±	0.4	5.5	±	0.0	18.0	±	0.1			
BaA	<MQL			1.1	±	0.0	1.1	±	0.0	<MQL					

**Table 4:** Carcinogenic risk calculated for rocket samples (crops irrigated using drinking water, treated wastewater and a commercial rocket).

	**RW1**	**RW2**	**RW3**	**RW4**	**RW5**
Cancer Risk	1.3 × 10^−8^	1.3 × 10^−8^	2.0 × 10^−8^	9.4 × 10^−9^	7.0 × 10^−9^
	RW6	RW7	RW8	C1	
Cancer Risk	7.2 × 10^−9^	2.4 × 10^−8^	2.3 × 10^−8^	1.6 × 10^−8^	

## Data Availability

The original contributions presented in this study are included in the article or Supplementary Material. Further inquiries can be directed to the corresponding authors.

## Supplementary Materials

The following supporting information can be downloaded at: https://www.mdpi.com/article/10.3390/foods14172963/s1, Figure S1. Effect of pH on the initial extraction step. Partitioning of green pigments into the water phase at pH 13.5 is clearly evident. Table S1. List of all 32 target analytes investigated in this study, including 15 PAHs, 3 N-PAHs, and 14 PCBs. For each compound, the table reports the compound name, abbreviation, molecular formula, molecular weight and MS quantifier ion (m/z). Table S2. Gas chromatographic oven temperature program and mass spectrometric acquisition parameters used for the determination of PAHs and PCBs in rocket samples. MS detection was carried out in electron impact (EI) ionization mode using selected ion monitoring (SIM), with quantifier ions listed in Table S1.
