# Supplementary material for: Residues of Priority Organic Micropollutants in Eruca vesicaria (Rocket) Irrigated by Reclaimed Wastewater: Optimization of a QuEChERS SPME-GC/MS Protocol and Risk Assessment
Source: Foods. 2025 Aug 25;14(17):2963. doi: 10.3390/foods14172963 (PMC12428525; doi:10.3390/foods14172963)
Supplement: Supplementary file 1 [file foods-14-02963-s001.zip › foods-3816177-supplementary.pdf]

Supplementary Material.

Residues of Priority Organic Micropollutants in *Eruca vesicaria* (Rocket) Irrigated by Reclaimed Wastewater: Optimization of a QuEChERS SPME-GC/MS Protocol and Risk Assessment

Luca Rivoira [1,*], Simona Di Bonito [1], Veronica Libonati [1], Massimo Del Bubba [2], Mihail Simion Beldean-Galea [3] and Maria Concetta Bruzzoniti [1,*]

[1] Department of Chemistry, Università degli Studi di Torino, Via Pietro Giuria 7, 10125 Torino, Italy
[2] Department of Chemistry "Ugo Schiff", Università degli Studi di Firenze, Via della Lastruccia 13, 50019 Sesto Fiorentino, Italy
[3] Faculty of Environmental Science and Engineering, Babeş-Bolyai University, 30 Fântânele Street, 400294 Cluj-Napoca, Romania
* Correspondence: luca.rivoira@unito.it (L.R.); mariaconcetta.bruzzoniti@unito.it (M.C.B.)

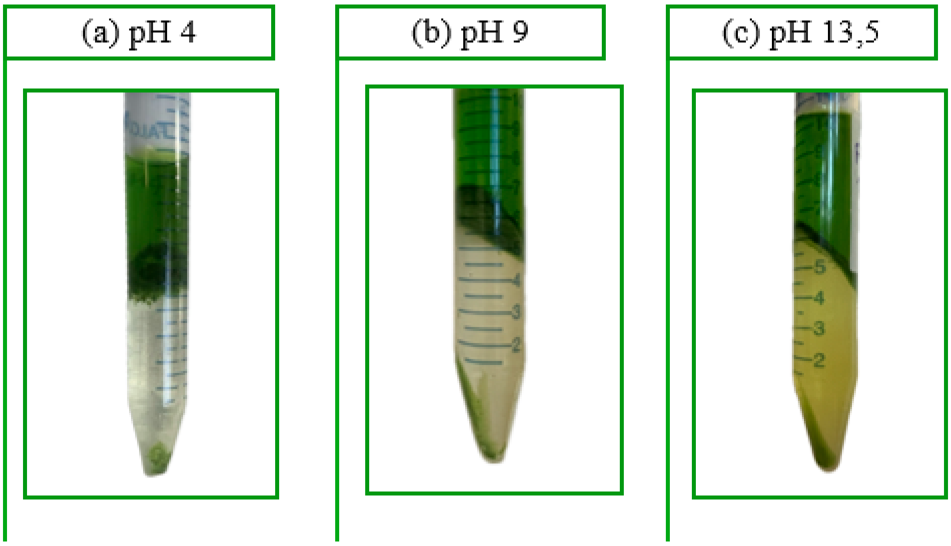

Figure S1. Effect of pH on the initial extraction step. Partitioning of green pigments into the water phase at pH 13.5 is clearly evident.

Table S1. List of all 32 target analytes investigated in this study, including 15 PAHs, 3 N-PAHs, and 14 PCBs. For each compound, the table reports the compound name, abbreviation, molecular formula, molecular weight and MS quantifier ion (m/z).

Class	Compound Name	Abbreviation	Molecular Formula	Molecular Weight (g/mol)	MS quantifier ion (m/z)
PAH	Acenaphthylene	AcPY	C12H8	152.2	152
PAH	Acenaphthene	AcPh	C12H10	154.21	152
PAH	Fluorene	Flu	C13H10	166.22	166
PAH	Phenanthrene	Phe	C14H10	178.23	178
PAH	Anthracene	Ant	C14H10	178.23	178
PAH	Fluoranthene	Flth	C16H10	202.25	202
PAH	Pyrene	Pyr	C16H10	202.25	202
PAH	Benzo[a]anthracene	BaA	C18H12	228.29	228
PAH	Chrysene	Chr	C18H12	228.29	228
PAH	Benzo[b]fluoranthene	BbFl	C20H12	252.31	252
PAH	Benzo[k]fluoranthene	BkFl	C20H12	252.31	252
PAH	Benzo[a]pyrene	BaP	C20H12	252.31	252
PAH	Indeno[1,2,3-cd]pyrene	Ind	C22H12	276.34	276
PAH	Dibenzo[a,h]anthracene	DBA	C22H14	278.35	278
PAH	Benzo[ghi]perylene	BP	C22H12	276.34	276
N-PAH	1-Nitronaphthalene	1-N-Naph	C10H7NO2	173.17	173
N-PAH	2-Nitrofluorene	2-N-Flu	C13H9NO2	211.2	211
N-PAH	1-Nitropyrene	1-N-Pyr	C16H9NO2	247.24	247
PCB	PCB 11	PCB 11	C12H5Cl5	326.4	222
PCB	PCB 15	PCB 15	C12H4Cl6	360.9	222
PCB	PCB 28	PCB 28	C12H10Cl2	257	186
PCB	PCB 52	PCB 52	C12H8Cl4	291	292
PCB	PCB 101	PCB 101	C12H7Cl5	325.4	292
PCB	PCB 81	PCB 81	C12H6Cl4	290	254
PCB	PCB 118	PCB 118	C12H5Cl5	326.4	326
PCB	PCB 123	PCB 123	C12H4Cl6	326.4	326
PCB	PCB 138	PCB 138	C12H4Cl6	360.9	360
PCB	PCB 153	PCB 153	C12H5Cl5	360.9	360
PCB	PCB 167	PCB 167	C12H3Cl7	394.4	360
PCB	PCB 180	PCB 180	C12H4Cl6	394.4	360
PCB	PCB 169	PCB 169	C12H3Cl7	394.4	394
PCB	PCB 189	PCB 189	C12H2Cl8	428.8	394

Table S2. Gas chromatographic oven temperature program and mass spectrometric acquisition parameters used for the determination of PAHs and PCBs in rocket samples. MS detection was carried out in electron impact (EI) ionization mode using selected ion monitoring (SIM), with quantifier ions listed in Table S1.

Parameter	Value
**Oven start temperature**	40 °C
**Hold time at start temperature**	2 min
**Ramp 1**	12 °C/min to 176 °C
**Ramp 2**	5 °C/min to 196 °C
**Hold time after Ramp 2**	3 min
**Ramp 3**	12 °C/min to 224 °C
**Hold time after Ramp 4**	3 min
**Ramp 5**	7 °C/min to 270 °C
**Hold time after Ramp 5**	3 min
**Final Ramp**	5 °C/min to 300 °C
**Final hold time**	10 min
**Injection volume**	2 µL
**Injection mode**	Pulsed splitless
**Injection pressure**	40 psi for 2 min
**Carrier gas**	Helium, 1.0 mL/min
**Column type**	(5%-Phenyl)-methylpolysiloxane (DB-5 ms)
**Column dimensions**	30 m — 0.25 mm — 0.25 µm
**MS ionization mode**	Electron Impact (EI) at 70 eV
**Acquisition mode**	Selected Ion Monitoring (SIM)
**Quantifier ions (m/z)**	See Table S1
**Software**	Agilent ChemStation
